# Clinical effectiveness of 0.018-inch vs. 0.022-inch bracket slot size in fixed orthodontic treatment: a systematic review and critical appraisal of the evidence

**DOI:** 10.3389/froh.2026.1862036

**Published:** 2026-07-08

**Authors:** Franz Tito Coronel-Zubiate, Heber Isac Arbildo-Vega, Fredy Hugo Cruzado-Oliva, Rubén Aguirre-Ipenza, Hernán Vásquez-Rodrigo, Sara Antonieta Luján-Valencia, Joan Manuel Meza-Málaga, Eduardo Luján-Urviola, Carlos Alberto Farje-Gallardo, Tania Belú Castillo-Cornock

**Affiliations:** 1 Faculty of Health Sciences, Universidad Nacional Toribio Rodríguez de Mendoza de Amazonas, Chachapoyas, Peru; 2 Postgraduate School, Universidad Nacional Toribio Rodríguez de Mendoza de Amazonas, Chachapoyas, Peru; 3 Faculty of Dentistry, Dentistry School, Universidad San Martín de Porres, Chiclayo, Peru; 4 Faculty of Human Medicine, Human Medicine School, Universidad San Martín de Porres, Chiclayo, Peru; 5 Faculty of Stomatology, Stomatology School, Universidad Nacional de Trujillo, Trujillo, Peru; 6 Faculty of Health Sciences, Universidad Continental, Lima, Peru; 7 Faculty of Health Sciences, Dentistry School, Universidad Norbert Wiener, Lima, Peru; 8 Postgraduate School, Universidad Católica de Santa María, Arequipa, Peru; 9 Faculty of Dentistry, Dentistry School, Universidad Católica de Santa María, Arequipa, Peru; 10 Faculty of Dentistry, Andina Néstor Cáceres Velásquez University, Juliaca, Peru

**Keywords:** 0.018-inch bracket, 0.022-inch bracket, bracket slot size, orthodontic appliances, orthodontic brackets, systematic review, treatment efficiency

## Abstract

**Background:**

Dentofacial malocclusion is a global public health concern and the third most prevalent oral condition after caries and periodontal disease. This systematic review evaluated the impact of bracket slot size (0.018 vs. 0.022-inch) on clinical efficacy (quality of finishing), tooth movement efficiency (treatment duration and alignment speed), and biosafety in patients treated with fixed orthodontic appliances.

**Material and methods:**

Randomized clinical trials were identified through comprehensive searches in PubMed, Cochrane Library, Scielo, Scopus, Web of Science, Embase, and Google Scholar up to January 2026. Studies comparing 0.018- and 0.022-inch slot brackets and reporting clinical effectiveness, efficiency, or biosafety outcomes were included. Risk of bias was assessed using RoB 2.0, and certainty of evidence was evaluated with the GRADE approach. Due to clinical and methodological heterogeneity among included studies, a statistically and clinically meaningful meta-analysis was not feasible, and results were synthesized narratively, incorporating a structured critical appraisal of the available evidence.

**Results:**

Of 835 records screened, nine RCTs met the inclusion criteria. No significant clinical differences were observed between bracket slot sizes regarding overall treatment duration, leveling efficiency, or final treatment quality. Although the 0.018-inch system required one fewer visit to complete alignment, this difference lacked clinical relevance. No significant differences in biological risks were identified. Higher comfort and improved quality of life were reported with 0.022-inch low-friction systems. Evidence certainty was low to very low for most outcomes.

**Conclusions:**

Based on current evidence, no clinically relevant differences exist between 0.018- and 0.022-inch slot brackets across most evaluated orthodontic outcomes. The overall certainty of evidence is low, and findings should be interpreted with caution. This review provides a structured critical appraisal of the available data, which may assist clinicians in making evidence-based decisions regarding bracket selection.

**Systematic Review Registration:**

https://www.crd.york.ac.uk/PROSPERO/view/CRD42024509885, PROSPERO CRD42024509885.

## 1 Introduction

Dentofacial malocclusion represents a significant global public health concern, with recent systematic reviews reporting a high and variable prevalence of orthodontic malocclusions among children and adolescents worldwide (1). Epidemiological studies report that between 11% and 93% of the population present some degree of malocclusion, with approximately 15% of adults experiencing functional or psychosocial consequences (2, 3). Fixed orthodontic appliances remain the most widely used treatment modality; however, prolonged treatment duration increases the risk of adverse effects such as root resorption, enamel demineralization, and reduced patient compliance (4).

Among the technical variables influencing treatment mechanics, bracket slot dimension has generated sustained clinical debate. Historically, the 0.022-inch slot was introduced to accommodate gold archwires, whereas the 0.018-inch slot later emerged to optimize the mechanical properties of stainless steel (5). Although the dimensional difference is minimal, it modifies the wire–bracket interaction and may affect torque expression, frictional resistance, and biomechanical efficiency (6, 7).

The 0.018-inch system has been associated with earlier torque expression and improved control during intermediate treatment stages, while the 0.022-inch system is considered advantageous for sliding mechanics and space closure due to increased clearance and versatility in archwire sequencing (4, 8). Despite these theoretical considerations, clinical evidence regarding treatment duration, alignment efficiency, finishing quality, and biological risks remains inconsistent and heterogeneous (9, 10).

Furthermore, discrepancies between nominal and actual slot dimensions reported by manufacturers may influence torque expression and treatment predictability, adding complexity to clinical decision-making. Consequently, slot selection is frequently based on personal preference rather than robust comparative evidence.

Although a recent systematic review by Rizk et al. (4) synthesized the available evidence on bracket slot size, important uncertainties remain regarding the methodological quality, certainty, and consistency of the reported outcomes. In addition, differences in study design, treatment protocols, outcome definitions, and follow-up periods have contributed to substantial clinical and methodological heterogeneity across the literature. Therefore, a critical appraisal of the available evidence focusing on randomized clinical trials is warranted to better understand the strengths, limitations, and certainty of current knowledge in this field.

Therefore, the objective of this systematic review was to evaluate the impact of bracket slot size (0.018 vs. 0.022-inch) on clinical efficacy, tooth movement efficiency, and biosafety in patients treated with fixed orthodontic appliances.

## 2 Materials and methods

### 2.1 Study design, research question and search strategy

This systematic review was conducted and reported in accordance with the PRISMA 2020 guidelines (11). The protocol was developed following PRISMA-P recommendations and registered in PROSPERO (12) under registration number CRD42024509885. Ethical approval was not required due to the nature of the study.

The focused question was structured using the PICO framework as follows: In patients undergoing orthodontic treatment with fixed appliances, what is the impact of bracket slot size (0.018-inch vs. 0.022-inch) on clinical effectiveness, tooth movement efficiency, and biological safety?

The population included patients treated with fixed orthodontic appliances. The intervention consisted of appliances using 0.018-inch bracket slots, defined as the slot dimension where the archwire is inserted to control tooth movement, while the comparator included 0.022-inch bracket slots. Outcomes assessed were the number of visits, treatment duration (overall and by stages), leveling and alignment efficiency, quality of treatment outcomes (ABO CR-Eval and PAR index), incisor angulation (U1-PP and L1-MP), orthodontically induced inflammatory root resorption, anchorage loss, tooth length changes, pain perception, and quality of life.

An electronic search was performed in PubMed, Cochrane Library, Scopus, Web of Science, Embase, and Scielo, complemented by gray literature searches in Google Scholar, OpenAIRE, and ProQuest Dissertations and Theses, including all records published up to January 2026. The reference lists of included studies were also screened. All records were imported into Zotero® (Center for History and New Media, Virginia, USA), and duplicates were removed. Detailed search strategies for each database are presented in Supplementary Material 1.

### 2.2 Study selection and data extraction

Study selection was performed using Rayyan® (Qatar Computing Research Institute, Qatar). Two reviewers independently screened titles, abstracts, and full texts, with disagreements resolved by a third reviewer.

Randomized clinical trials evaluating the impact of 0.018-inch vs. 0.022-inch bracket slot size in patients treated with fixed orthodontic appliances were included, without language or date restrictions. Observational studies, reviews, case reports, *in vitro* or animal studies, unpublished data, and duplicate publications were excluded.

Data were independently extracted by two reviewers (F.T.C.-Z. and F.H.C.-O.) using a standardized data collection form, collecting study characteristics and relevant clinical, radiographic, and patient-reported outcomes, including treatment duration, number of visits, orthodontic indices (ABO CR-Eval, PAR), incisor angulation, root resorption, anchorage loss, tooth length changes, pain, and quality of life.

### 2.3 Risk of bias (RoB) assessment

The RoB of the included studies was independently assessed by two calibrated authors (J.M.M.-M. and E.L.-U.) (k = 0.98) using the Cochrane Group's RoB 2.0 tool (13) and all disagreements were resolved by discussion with a third reviewer (C.F.). According to this tool, clinical trials are evaluated in five domains: randomization process, deviations from planned interventions, missing outcome data, outcome measurement, and selection of the results report; to later be classified as: high risk of bias, bias with some concerns, or low risk of bias.

### 2.4 Data synthesis

Due to substantial clinical and methodological heterogeneity among included randomized clinical trials, particularly in outcome definitions, follow-up periods, intervention protocols, and reporting formats, a statistically and clinically meaningful meta-analysis was not feasible.

### 2.5 Certainty of evidence (GRADE)

The certainty of evidence for each outcome was assessed using the Grading of Recommendations Assessment, Development and Evaluation (GRADE) approach through the GRADEpro GDT software (McMaster University and Evidence Prime Inc., Canada).

## 3 Results

### 3.1 Review and selection of primary studies

**Figure 1:**
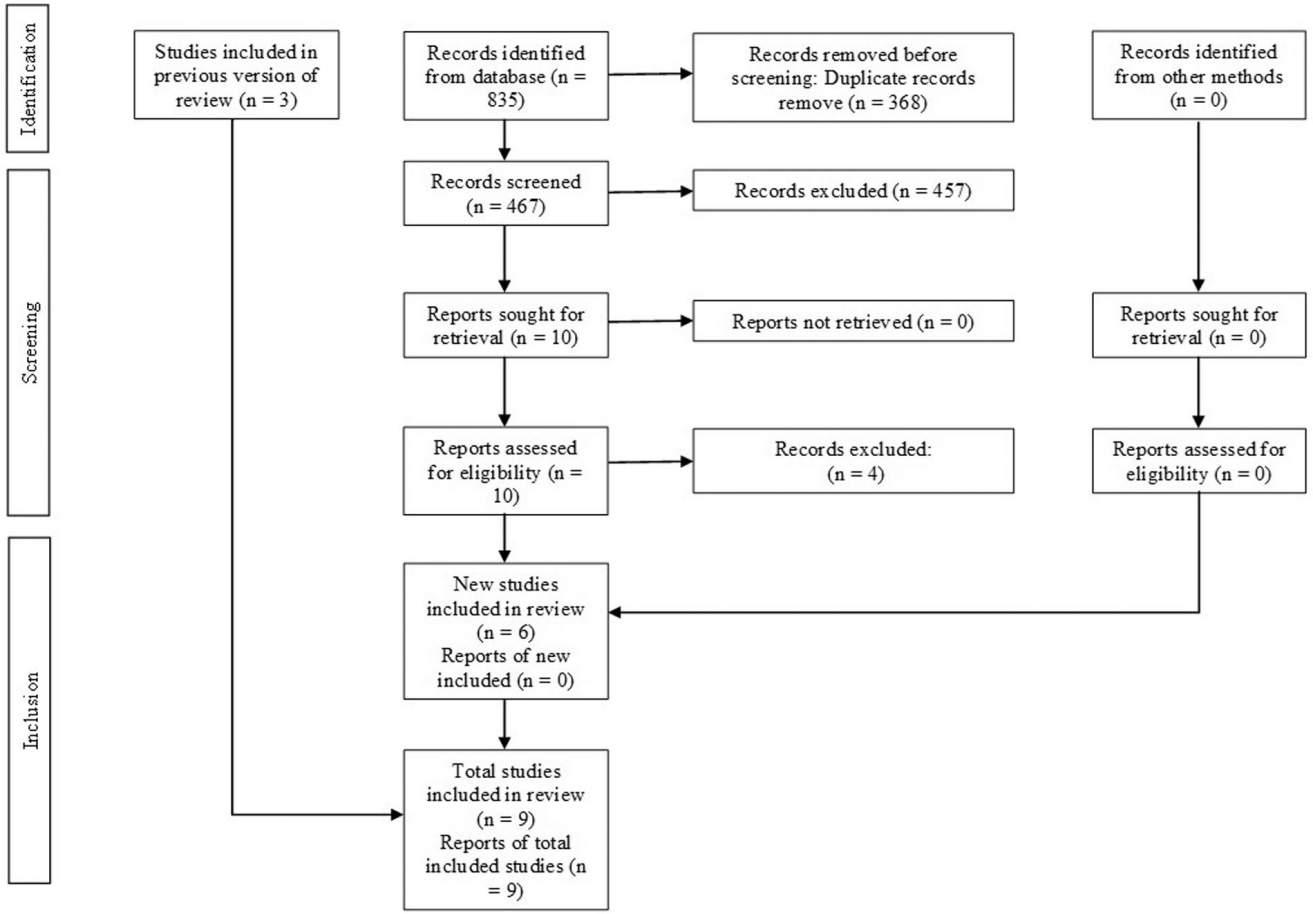
PRISMA flow diagram showing the selection process of studies included in the systematic review, from initial identification to final inclusion.

A total of 835 records were identified through electronic database searches. After removal of duplicates, 467 records remained for screening. Title and abstract evaluation resulted in 10 studies eligible for full-text assessment. Four studies were excluded after full-text review, while three additional studies were identified through manual screening of reference lists. Ultimately, nine randomized clinical trials were included in the qualitative synthesis. Reasons for exclusion are detailed in Supplementary Material 2, and the study selection process is presented in Figure 1.

### 3.2 Review and characteristics of included studies

Nine randomized clinical trials (RCTs) (14–22) were included (detailed in Supplementary Material 3), of which one followed a crossover design (21). Sample sizes ranged from 14 to 153 participants. Six studies reported mean ages between 12.3 and 24.4 years (15–18, 21, 22), while eight studies described an overall age range of 10–40 years (14–18, 20–22). Follow-up periods varied from 4 to 29.3 months (Supplementary Material 3).

The studies were conducted in Egypt (14), Spain (15), the United Kingdom (16–19), India (20), Austria (21), and the Netherlands (22). Treatment duration outcomes were reported in two studies for the levelling and alignment stage (16, 20) and in two studies for overall treatment time (15, 21). Individual clinical and radiographic outcomes—including number of visits (14), pain and quality of life (15), levelling and alignment efficiency (19), working and finishing stage duration and ABO CR-Eval (16), PAR index and incisor angulation (U1–PP, L1–MP) (17), orthodontically induced inflammatory root resorption (18), anchorage loss (19), maxillary and mandibular shape changes (21), and tooth length reduction and prevalence of resorption (22)—were each reported by single studies (Supplementary Material 3).

### 3.3 Risk of bias analysis of studies

Three randomized clinical trials were judged to have a high risk of bias (14, 21, 22), while six were assessed as having a low risk of bias (15–20).

### 3.4 Synthesis of results

Overall, the available evidence did not demonstrate consistent clinically relevant differences between 0.018-inch and 0.022-inch bracket slot systems regarding treatment efficiency, treatment quality, biological response, or anchorage control. However, interpretation of the findings should consider the substantial clinical and methodological heterogeneity among the included studies, including differences in malocclusion characteristics, treatment protocols, appliance systems, ligation methods, and outcome assessment procedures.

One randomized clinical trial (14) reported a statistically significant reduction in the number of visits required to achieve alignment with 0.018-inch brackets compared with 0.022-inch brackets (*p* = 0.017). However, the observed difference of one visit was considered clinically negligible, and the study was limited to mild-to-moderate non-extraction cases.

Regarding treatment duration, Yassir et al. (15) found no significant differences in overall treatment time between bracket slot systems despite an attrition rate of 18.1%. Regression analysis suggested that treatment duration was more strongly associated with patient- and management-related factors than with slot dimension itself. Similarly, Bhardwaj et al. (19) reported no significant differences in alignment efficiency between 0.018-inch and 0.022-inch slot systems. Celar et al. (20) also reported no significant differences in dental arch shape changes between the compared appliance systems; However, interpretation of slot size effects in this study is limited because bracket type, ligation mode, and treatment mechanics differed simultaneously.

With respect to treatment quality, Yassir et al. (16) reported comparable ABO CR-EVAL scores, PAR reduction, and incisor inclination changes between treatment groups, suggesting similar clinical effectiveness regardless of bracket slot size.

Patient-reported outcomes showed mixed findings. Curto et al. (14) reported lower pain levels and better oral health-related quality of life in patients treated with a low-friction 0.022-inch system, although peak pain occurred within 24–48 hours in all groups. In contrast, El-Angbawi et al. (17) found no significant differences in pain perception between the evaluated bracket systems.

Biological outcomes were generally comparable across studies. El-Angbawi et al. (17) reported no significant differences in orthodontically induced inflammatory root resorption (OIIRR) between 0.018-inch and 0.022-inch slot systems. Likewise, Reukers et al. (21) found no significant differences in the prevalence or degree of apical root resorption between the appliance systems evaluated in their randomized clinical trial. Similarly, Yassir et al. (18) observed no significant differences in molar anchorage loss between 0.018-inch and 0.022-inch bracket systems.

Taken together, the available evidence suggests that bracket slot size alone is unlikely to be a major determinant of treatment efficiency, treatment quality, or biological response. However, interpretation of these findings should consider the influence of additional biomechanical and clinical factors that varied among studies and may have contributed to the observed outcomes.

### 3.5 GRADE analysis

The certainty of evidence was rated as very low for number of visits, overall treatment duration, maxillary and mandibular shape changes, tooth length reduction, and prevalence of resorption. Low certainty was assigned to pain perception, quality of life, duration of the leveling and alignment stage, working and finishing stage duration, ABO CR-Eval, PAR index, incisor angulation (U1–PP and L1–MP), orthodontically induced inflammatory root resorption (OIIRR), and anchorage loss (Supplementary Material 4). The downgrading of evidence certainty was mainly related to study limitations, small sample sizes, outcome-specific imprecision, and the limited number of available studies for several outcomes.

## 4 Discussion

Our results indicate that there are no clinically relevant differences between 0.018-inch and 0.022-inch bracket slot systems in terms of clinical efficacy or biological safety. Total treatment duration and the alignment, leveling, and finishing phases were comparable between both systems (16, 17), as were final occlusion quality and patient-reported outcomes, including pain and quality of life (17, 18). While these findings are broadly consistent with those reported by Rizk et al. (4), important methodological differences distinguish the present review. Unlike the previous synthesis, which combined randomized and non-randomized evidence and focused primarily on treatment duration, the present review restricted inclusion to randomized clinical trials and evaluated a broader range of clinical, biological, and patient-reported outcomes. Furthermore, outcome-specific assessments of risk of bias and certainty of evidence were performed using RoB 2.0 and GRADE, providing a more detailed appraisal of the strengths, limitations, and consistency of the available evidence. Overall, although no substantial advantage of one system over the other was identified, the low certainty of evidence highlights the need for cautious interpretation. In addition, important sources of clinical heterogeneity were identified across studies, including differences in baseline malocclusion characteristics, treatment modality, and extraction protocols. Furthermore, some investigations employed split-mouth designs, in which different appliance systems were applied to opposite hemiarches. In such situations, biomechanical interactions between both sides may limit the ability to isolate the independent effect of bracket slot size on treatment outcomes.

These findings suggest that bracket slot size is unlikely to be a primary determinant of treatment outcomes in routine clinical orthodontic practice. Yassir et al. (15) reported similar total treatment durations between 0.018-inch and 0.022-inch slots, with comparable orthodontic quality (assessed using ABO CR-Eval and PAR) and patient aesthetic perception (17). Within the same cohort, El-Angbawi et al. (17) found that the severity of orthodontically induced inflammatory root resorption (OIIRR) and patient-reported pain were not influenced by slot size. Additional evidence from the broader literature supports these findings.

Some studies reported minor contextual differences between bracket systems. For instance, the 0.018-inch slot completed initial alignment on average one visit sooner than the 0.022-inch slot (14). Other reports indicate less pain with the 0.022-inch slot (15), whereas Amditis and Smith (22) observed a trend toward shorter treatment duration with the 0.018-inch slot. However, these findings have not been consistently reproduced across subsequent studies and should be interpreted with caution. Similarly, Detterline et al. (7) reported minor differences in treatment quality assessed using the ABO Objective Grading System. However, these variations were heterogeneous and of limited clinical relevance and do not contradict the overall conclusion that neither slot system demonstrates a clear and consistent clinical advantage.

From a biomechanical perspective, it has been suggested that slot sizes may influence treatment progression in opposite ways: the 0.022-inch slot allows greater initial clearance and facilitates the use of thicker archwires with lower friction, whereas the 0.018-inch slot promotes faster engagement and earlier torque expression (4, 9, 24). In clinical practice, these theoretical differences tend to offset each other, making slot selection largely a matter of clinician preference rather than significant technical advantage (23).

The literature review reveals important methodological limitations. Substantial clinical and methodological heterogeneity among the included randomized clinical trials prevented a statistically robust meta-analysis. Sources of heterogeneity included differences in malocclusion characteristics, extraction and non-extraction treatment protocols, bracket systems (conventional, low-friction, and different ligation modes), which may independently influence treatment mechanics and clinical outcomes, as well as variations in outcome definitions, follow-up periods, and measurement methods. Unfortunately, these variables were not reported consistently across studies, precluding subgroup analyzes according to malocclusion type or treatment modality. In addition, several outcomes were reported by single studies, limiting opportunities for quantitative synthesis and reducing comparability across investigations. Furthermore, several publications from the United Kingdom originated from the same multicenter randomized clinical trial and reported different outcomes from the same participant cohort. Although no double-counting of participants occurred because a meta-analysis was not performed, this overlap may increase the apparent volume of evidence and should be considered when interpreting the independence of the available findings. These factors precluded the performance of a statistically and clinically meaningful meta-analysis. Moreover, some trials presented a high risk of bias, and the GRADE assessment indicated very low certainty for several outcomes and low certainty for others (7, 14, 18). The downgrading for very serious imprecision was primarily related to the limited number of available studies, small sample sizes, and the fact that several outcomes were reported by single trials. Therefore, these findings should be interpreted with caution.

Clinically, the results suggest that slot size choice does not substantially alter orthodontic treatment outcomes. Orthodontists may select either 0.018-inch or 0.022-inch slots based on preference, experience, or material availability, without expecting significant differences in efficiency or safety, consistent with recent randomized clinical evidence (4, 23, 25). However, given the low to very low certainty of evidence for most outcomes and the heterogeneity among included trials, these findings should be interpreted with caution. Future research should prioritize adequately powered multicenter randomized clinical trials using standardized outcome measures, uniform bracket prescriptions, clearly defined treatment protocols, and consistent follow-up periods. Particular attention should be given to clinically relevant outcomes such as treatment duration, treatment quality, root resorption, anchorage loss, pain perception, oral health-related quality of life, and patient satisfaction. Future studies should also control for potential confounding factors, including extraction vs. non-extraction protocols, bracket design (conventional vs. self-ligating systems), and malocclusion severity, to better determine whether bracket slot size independently influences treatment outcomes.

In summary, the evidence indicates that the choice of slot size has minimal practical impact on orthodontic outcomes. After analyzing the nine randomized clinical trials included in this review, it can be concluded that there are no significant clinical differences between 0.018-inch and 0.022-inch slot brackets in terms of overall treatment duration, alignment and leveling efficiency, or final orthodontic outcomes evaluated by excellence indices. Nevertheless, the limited certainty of the available evidence highlights the need for cautious interpretation and further high-quality research before definitive clinical recommendations can be established. Although the 0.018-inch system may show a slight statistical advantage in the number of visits required to complete alignment (7 vs. 8 visits), this difference is clinically negligible. Both slot sizes demonstrate comparable biological effects, including root resorption and anchorage loss, indicating that patient safety is not influenced by slot size. Therefore, the choice between these systems should primarily depend on the clinician's technical preference and material availability, within the limitations of the current evidence base.

## 5 Conclusions

Within the limitations of the available evidence, no clinically meaningful differences were identified between 0.018-inch and 0.022-inch bracket slot systems. Given the low to very low certainty of evidence, these findings should be interpreted with caution. Further high-quality randomized clinical trials are required.

## Data Availability

The original contributions presented in the study are included in the article/Supplementary Material, further inquiries can be directed to the corresponding author.

## References

[1] De Ridder L Aleksieva A Willems G Declerck D de Llano-Pérula MC. Prevalence of orthodontic malocclusions in healthy children and adolescents: a systematic review. Int J Environ Res Public Health. (2022) 19:7446. 10.3390/ijerph19127446 35742703 PMC9223594

[2] Hung M Zakeri G Su S Mohajeri A. Profile of orthodontic use across demographics. Dent J (Basel). (2023) 11:291. 10.3390/dj11120291 38132429 PMC10742803

[3] Borzabadi-Farahani A Eslamipour F Asgari I. Association between orthodontic treatment need and caries experience. Acta Odontol Scand. (2011) 69:2–11. 10.3109/00016357.2010.516732 20923258

[4] Rizk MZ Mohammed H Samy J McIntyre G Bearn D. Duration of fixed appliance treatment using 0.018-inch slot versus 0.022-inch slot brackets: a systematic review and meta-analysis. Eur J Orthod. (2025) 47:cjaf082. 10.1093/ejo/cjaf082 41091659 PMC12525148

[5] Ruchi S Vijay A Kumar AM Amit S. 0.018” versus 0.022” bracket slot systems in orthodontics: a review. Unique J Med Dent Sci. (2014) 2:84–7.

[6] Vieira EP Watanabe BSD Pontes LF Mattos JNF Maia LC Normando D. The effect of bracket slot size on the effectiveness of orthodontic treatment: a systematic review. Angle Orthod. (2018) 88:100–6. 10.2319/031217-185.1 28949767 PMC8315718

[7] Detterline DA Isikbay SC Brizendine EJ Kula KS. Clinical outcomes of 0.018-inch and 0.022-inch bracket slot using the ABO objective grading system. Angle Orthod. (2010) 80:528–32. 10.2319/060309-315.1 20050748 PMC8985707

[8] Epstein MB Epstein JZ. Benefits and rationale of differential bracket slot sizes: the use of 0.018-inch and 0.022-inch slot sizes within a single bracket system. Angle Orthod. (2002) 72:1–2. 10.1043/0003-3219(2002)072<0001:BARODB>2.0.CO;2 11843268 10.1043/0003-3219(2002)072<0001:BARODB>2.0.CO;2

[9] Papageorgiou SN Sifakakis I Doulis I Eliades T Bourauel C. Torque efficiency of square and rectangular archwires into 0.018 and 0.022 in. conventional brackets. Prog Orthod. (2016) 17:5. 10.1186/s40510-016-0118-0 26780465 PMC4715034

[10] Shamseer L Moher D Clarke M Ghersi D Liberati A Petticrew M. Preferred reporting items for systematic review and meta-analysis protocols (PRISMA-P) 2015: elaboration and explanation. Br Med J. (2015) 350:g7647. 10.1136/bmj.g7647 25555855

[11] Booth A Clarke M Ghersi D Moher D Petticrew M Stewart L. An international registry of systematic-review protocols. Lancet. (2011) 377:108–9. 10.1016/S0140-6736(10)60903-8 20630580

[12] Sterne JAC Savović J Page MJ Elbers RG Blencowe NS Boutron I. Rob 2: a revised tool for assessing risk of bias in randomised trials. Br Med J. (2019) 366:l4898. 10.1136/bmj.l4898 31462531

[13] Farahat MBM Aboelmahasen MMF Elghetany R. The effects of the twin arch bracket system in class 1 orthodontic patients for a control of orthodontic tooth movement: a randomized controlled clinical trial. APOS Trends Orthod. (2025) 15:205–17. 10.25259/APOS_211_2024

[14] Curto A Albaladejo A Montero J Alvarado-Lorenzo M Garcovich D Alvarado-Lorenzo A. A prospective randomized clinical trial to evaluate the slot size on pain and oral health-related quality of life (OHRQoL) in orthodontics during the first month of treatment with conventional and low-friction brackets. Appl Sci. (2020) 10:7136. 10.3390/app10207136

[15] Yassir YA El-Angbawi AM McIntyre GT Revie GF Bearn DR. A randomized clinical trial of the effectiveness of 0.018-inch and 0.022-inch slot orthodontic bracket systems: part 1-duration of treatment. Eur J Orthod. (2019) 41:133–42. 10.1093/ejo/cjy037 30007300

[16] Yassir YA El-Angbawi AM McIntyre GT Revie GF Bearn DR. A randomized clinical trial of the effectiveness of 0.018-inch and 0.022-inch slot orthodontic bracket systems: part 2-quality of treatment. Eur J Orthod. (2019) 41:143–53. 10.1093/ejo/cjy038 30007333

[17] El-Angbawi AM Yassir YA McIntyre GT Revie GF Bearn DR. A randomized clinical trial of the effectiveness of 0.018-inch and 0.022-inch slot orthodontic bracket systems: part 3-biological side-effects of treatment. Eur J Orthod. (2019) 41:154–64. 10.1093/ejo/cjy039 30007330

[18] Yassir YA McIntyre GT El-Angbawi AM Bearn DR. Does anchorage loss differ with 0.018-inch and 0.022-inch slot bracket systems? Angle Orthod. (2019) 89:605–10. 10.2319/081918-608.1 31013131 PMC8117205

[19] Bhardwaj P Sonar S Batra P. Alignment efficiency of standard versus tandem wire mechanics using conventional and self-ligating brackets: a pilot study. J Indian Orthod Soc. (2017) 51:103–9. 10.4103/jios.jios_208_16

[20] Celar AG Onodera K Bertl MH Astl E Bantleon H-P Sato S. Geometric morphometric evaluations of a randomized prospective split-mouth study on modes of ligation and reverse-curve mechanics. Orthod Craniofac Res. (2014) 17:158–69. 10.1111/ocr.12042 24720396

[21] Reukers EA Sanderink GC Kuijpers-Jagtman AM van’t Hof MA. Radiographic evaluation of apical root resorption with 2 different types of edgewise appliances. Results of a randomized clinical trial. J Orofac Orthop. (1998) 59:100–9. 10.1007/BF01340644 9577105

[22] Amditis C Smith LF. The duration of fixed orthodontic treatment: a comparison of two groups of patients treated using edgewise brackets with 0.018” and 0.022” slots. Aust Orthod J. (2000) 16:34–9. 11201958

[23] Eliades T. Bracket slot size selection: after all a matter of taste? Eur J Orthod. (2019) 41:223–4. 10.1093/ejo/cjy051 30184050

[24] Li Y Tang N Xu Z Feng X Yang L Zhao Z. Bidimensional techniques for stronger anterior torque control in extraction cases: a combined clinical and typodont study. Angle Orthod. (2012) 82:715–22. 10.2319/082811-550.1 22149540 PMC8845548

[25] Abouwafia O Majeed HA Fayed M Seif-Eldin NF. Comparison of alignment duration between 0.018” and 0.022” slot brackets in non-extraction orthodontic adult patients: a randomized clinical study. Future Dent J. (2024) 10:49–52. 10.54623/fdj.1018

