# Supplementary material for: Clinical effectiveness of 0.018-inch vs. 0.022-inch bracket slot size in fixed orthodontic treatment: a systematic review and critical appraisal of the evidence
Source: Front Oral Health. 2026 Jul 8;7:1862036. doi: 10.3389/froh.2026.1862036 (PMC13388385; doi:10.3389/froh.2026.1862036)
Supplement: Supplementary file 1 [file Table1.docx]

Supplementary Material 1. Search strategies for each database.

| **Database** | **Search strategy** | **Number of studies** |
| --- | --- | --- |
| Pubmed | (("Orthodontic Brackets"[MeSH Terms] OR "Orthodontic Appliances, Fixed"[MeSH Terms] OR "fixed appliance*"[Title/Abstract] OR "fixed orthodontic*"[Title/Abstract] OR "bracket*"[Title/Abstract]) AND ("0.018"[Title/Abstract] OR "0.022"[Title/Abstract] OR "slot size*"[Title/Abstract] OR "slot dimension*"[Title/Abstract])) AND ("randomized controlled trial"[pt] OR "controlled clinical trial"[pt] OR "randomized"[tiab] OR "placebo"[tiab] OR "randomly"[tiab] OR "clinical trial"[tiab]) | 109 |
| Cochrane Library | ([mh "Orthodontic Brackets"] OR [mh "Orthodontic Appliances, Fixed"] OR "fixed appliance":ti,ab,kw OR "fixed orthodontic":ti,ab,kw OR "brackets":ti,ab,kw ) AND ( "0.018":ti,ab,kw OR "0.022":ti,ab,kw OR "slot size":ti,ab,kw OR "slot dimension":ti,ab,kw ) | 208 |
| Scopus | (TITLE-ABS-KEY (("fixed appliance*" OR "fixed orthodontic*" OR "bracket*" OR "orthodontic bracket*")) AND TITLE-ABS-KEY (("0.018" OR "0.022" OR "slot size*" OR "slot dimension*")) AND TITLE-ABS-KEY (("randomized controlled trial" OR "random*" OR "clinical trial"))) | 124 |
| Web of Science | "fixed appliance*" OR "fixed orthodontic*" OR "brackets" OR "orthodontic bracket*" (Topic) AND "0.018" OR "0.022" OR "slot size*" OR "slot dimension*" (Topic) AND "randomized controlled trial" OR "random*" OR "clinical trial" (Topic) | 85 |
| Embase | ('orthodontic bracket'/exp OR 'fixed orthodontic appliance'/exp OR 'fixed appliance*':ti,ab OR 'fixed orthodontic*':ti,ab OR 'brackets':ti,ab) AND ('0.018':ti,ab OR '0.022':ti,ab OR 'slot size*':ti,ab OR 'slot dimension*':ti,ab) AND ('randomized controlled trial'/exp OR 'randomization'/exp OR 'controlled clinical trial'/exp OR 'double blind procedure'/exp OR 'single blind procedure'/exp OR random*:ti,ab OR factorial*:ti,ab OR crossover*:ti,ab OR (cross NEXT/1 over*):ti,ab OR placebo*:ti,ab OR (doubl* NEXT/1 blind*):ti,ab OR (singl* NEXT/1 blind*):ti,ab OR assign*:ti,ab OR allocat*:ti,ab OR volunteer*:ti,ab) | 170 |
| Scielo | (ti:("brackets" OR "fixed appliance" OR "aparatología fija" OR "aparelho fixo")) AND (ab:("0.018" OR "0.022" OR "slot size" OR "tamaño de ranura" OR "tamanho da ranhura")) | 13 |
| OpenAIRE | (title:("orthodontic" AND "slot") OR description:("0.018" AND "0.022" AND "randomized")) | 0 |
| Proquest Dissertations and Theses | TI,AB("fixed appliance*" OR "fixed orthodontic*" OR "orthodontic bracket*") AND TI,AB("0.018" NEAR/3 "0.022" OR "slot size*" OR "slot dimension*") AND TI,AB("random*" OR "clinical trial") | 4 |
| Google Scholar | "fixed orthodontic" "slot size" ("0.018" OR "0.022") "randomized" -"in vitro" | 122 |
