# Supplementary material for: Clinical effectiveness of 0.018-inch vs. 0.022-inch bracket slot size in fixed orthodontic treatment: a systematic review and critical appraisal of the evidence
Source: Front Oral Health. 2026 Jul 8;7:1862036. doi: 10.3389/froh.2026.1862036 (PMC13388385; doi:10.3389/froh.2026.1862036)
Supplement: Supplementary file 2 [file Table2.docx]

Supplementary Material 2. Reasons for exclusion of studies

| **Author** | **Reason for exclusion** |
| --- | --- |
| Farahat et al. (14) | Double-arch system |
| Cobb et al. (15) | Does not compare slots |
| Sobouti et al. (16) | Non-randomized study |
| Lia et al. (17) | Two-dimensional technique |
