# Supplementary material for: Clinical effectiveness of 0.018-inch vs. 0.022-inch bracket slot size in fixed orthodontic treatment: a systematic review and critical appraisal of the evidence
Source: Front Oral Health. 2026 Jul 8;7:1862036. doi: 10.3389/froh.2026.1862036 (PMC13388385; doi:10.3389/froh.2026.1862036)
Supplement: Supplementary file 3 [file Table3.docx]

Supplementary Material 3. Characteristics of included studies

| **Author(s)** | **Year** | **Type of study** | **Country** | **Number of patients (male/female)** | **Average age (range)** | **Follow-up** | **Groups** | **Number of patients per group** | **Outcomes** | |
| --- | --- | --- | --- | --- | --- | --- | --- | --- | --- | --- |
| Abouwafia et al. (17) | 2024 | RCT parallel | Egypt | 14 | (14-25) | 8 months | 0.018 | 7 | NV | 7 ± 0.58 |
|  |  |  |  |  |  |  | 0.022 | 7 | NV | 8 ± 0.58 |
| Curto et al. (18) | 2020 | RCT parallel | Spain | 120 (61/59) | 24.4 ± 11.4 (18-40) | 1 month | 0.018 | 30 | Pain | 1.3 ± 1.6 |
|  |  |  |  |  |  |  |  |  | QL | 3.8 ± 2.1 |
|  |  |  |  |  |  |  | 0.022 | 30 | Pain | 1.8 ± 1.9 |
|  |  |  |  |  |  |  |  |  | QL | 3 ± 1.9 |
|  |  |  |  |  |  |  | LF 0.018 | 30 | Pain | 1.6 ± 2.1 |
|  |  |  |  |  |  |  |  |  | QL | 4.5 ± 2.8 |
|  |  |  |  |  |  |  | LF 0.022 | 30 | Pain | 1.3 ± 1.8 |
|  |  |  |  |  |  |  |  |  | QL | 2.1 ± 1.4 |
| Yassir et al. (19) | 2019 | RCT parallel | United Kingdom | 153 (48/105) | 19.1 ± 8.5 (≥ 12) | 29.3 months | 0.018 | 77 | DL & AS | 11.8 ± 5 |
|  |  |  |  |  |  |  |  |  | DW & FS | 17.4 ± 9.1 |
|  |  |  |  |  |  |  |  |  | DOT | 29.3 ± 9.5 |
|  |  |  |  |  |  |  | 0.022 | 76 | DL & AS | 11.8 ± 6.2 |
|  |  |  |  |  |  |  |  |  | DW & FS | 19.4 ± 11.7 |
|  |  |  |  |  |  |  |  |  | DOT | 31.2 ± 12.3 |
| Yassir et al. (20) | 2019 | RCT parallel | United Kingdom | 153 (48/105) | 19.1 ± 8.5 (≥ 12) | NR | 0.018 | 77 | ABO CR-Eval | 34.7 ± 9.5 |
|  |  |  |  |  |  |  |  |  | PAR | 7.4 ± 5.1 |
|  |  |  |  |  |  |  |  |  | U1-PP | 110.2 ± 7.5 |
|  |  |  |  |  |  |  |  |  | L1-MP | 94.1 ± 7.9 |
|  |  |  |  |  |  |  | 0.022 | 76 | ABO CR-Eval | 34.5 ± 11 |
|  |  |  |  |  |  |  |  |  | PAR | 6 ± 4.4 |
|  |  |  |  |  |  |  |  |  | U1-PP | 110.2 ± 7.6 |
|  |  |  |  |  |  |  |  |  | L1-MP | 94.3 ± 7.9 |
| El-Angbawi et al. (21) | 2019 | RCT parallel | United Kingdom | 153 (48/105) | 19.1 ± 8.5 (≥ 12) | 9 months | 0.018 | 77 | OIIRR | 1.3 ± 0.95 |
|  |  |  |  |  |  |  | 0.022 | 76 | OIIRR | 1.09 ± 1.01 |
| Yassir et al. (22) | 2019 | RCT parallel | United Kingdom | 74 | NR | NR | 0.018 | 41 | ALR | 3.86 ± 2.15 |
|  |  |  |  |  |  |  |  |  | ALL | 3.3 ± 2.03 |
|  |  |  |  |  |  |  | 0.022 | 33 | ALR | 3.73 ± 1.87 |
|  |  |  |  |  |  |  |  |  | ALL | 3.47 ± 1.69 |
| Bhardwaj et al. (23) | 2017 | RCT parallel | India | 35 | (14-25) | 4 months | SL 0.018 | 7 | DL & AS | 105.86 ± 28.11 |
|  |  |  |  |  |  |  |  |  | L & A | 1.06 ± 0.98 |
|  |  |  |  |  |  |  | SL 0.018 Tandem | 7 | DL & AS | 126.21 ± 8.08 |
|  |  |  |  |  |  |  |  |  | L & A | 1.17 ± 0.52 |
|  |  |  |  |  |  |  | SL 0.022 | 7 | DL & AS | 90.29 ± 6.73 |
|  |  |  |  |  |  |  |  |  | L & A | 1.52 ± 1.09 |
|  |  |  |  |  |  |  | SL 0.022 Tandem | 7 | DL & AS | 127.86 ± 8.24 |
|  |  |  |  |  |  |  |  |  | L & A | 1.45 ± 1.09 |
|  |  |  |  |  |  |  | 0.022 | 7 | DL & AS | 92.5 ± 11.71 |
|  |  |  |  |  |  |  |  |  | L & A | 0.92 ± 0.54 |
| Celar et al. (24) | 2014 | RCT cross-over | Austria and Japan | 20 (4/16) | 22.5 ± 5.7 (14-36) | 9 months | 0.018 | 20 | SCM | 0.0194 |
|  |  |  |  |  |  |  |  |  | SCMb | 0.0187 |
|  |  |  |  |  |  |  | SL 0.022 | 20 | SCM | 0.0109 |
|  |  |  |  |  |  |  |  |  | SCMb | 0.0112 |
| Reukers et al. (25) | 1998 | RCT parallel | Netherlands | 61 | 12.3 ± 1.2 (10-15) | 1.7 years | 0.018 | 29 | DOT | 1.6 ± 0.5 |
|  |  |  |  |  |  |  |  |  | LTL | 7.5 ± 7.6 |
|  |  |  |  |  |  |  |  |  | PR | 16/29 |
|  |  |  |  |  |  |  | 0.022 | 32 | DOT | 1.8 ± 0.4 |
|  |  |  |  |  |  |  |  |  | LTL | 8.2 ± 6.4 |
|  |  |  |  |  |  |  |  |  | PR | 24/32 |

*RCT = Randomized clinical trial; LF = Low-friction; SL = Self‑ligating; NV = Number of visits; QL = Quality of life; DL & AS = Duration of levelling & alignment stage; DW & FS = Duration of working & finishing stage; DOT = Duration of overall treatment; ABO CR-Eval = The American Board of Orthodontics Model and Radiograph Evaluation - Quality of orthodontic treatment outcomes; PAR = Peer Assessment Rating - Severity of malocclusion and quality of the final treatment outcome; U1-PP = Angle formed between the longitudinal axis of the upper incisor (U1) and the palatal plane (PP); L1-PP = Angle between the longitudinal axis of the lower incisor (L1) and the mandibular plane (MP); OIIRR = Orthodontically induced inflammatory root resorption; ALR = Anchor loss right; ALL = Anchor loss left; L & A = Levelling & alignment; SCM = Shape Change Maxilla; SCMb = Shape Change Mandible; LTL = Loss of tooth length; PR = Prevalence of resorption*
