# Supplementary material for: Clinical effectiveness of 0.018-inch vs. 0.022-inch bracket slot size in fixed orthodontic treatment: a systematic review and critical appraisal of the evidence
Source: Front Oral Health. 2026 Jul 8;7:1862036. doi: 10.3389/froh.2026.1862036 (PMC13388385; doi:10.3389/froh.2026.1862036)
Supplement: Supplementary file 4 [file Table4.docx]

Supplementary Material 4. GRADE analysis of included studies.

| **Certainty assessment** | | | | | | | **№ of patients** | | **Certainty** |
| --- | --- | --- | --- | --- | --- | --- | --- | --- | --- |
| **№ of studies** | **Study design** | **Risk of bias** | **Inconsistency** | **Indirectness** | **Imprecision** | **Other considerations** | **slot 0.018-inch** | **slot 0.022-inch** |  |
| **Number of visits (follow-up: 8 months)** | | | | | | | | | |
| 1 | randomised trials | very serious | not serious | not serious | very serious | none | 7 | 7 | ⨁◯◯◯ Very low |
| **Pain (follow-up: 1 months)** | | | | | | | | | |
| 1 | randomised trials | not serious | not serious | not serious | very serious | none | 30 | 30 | ⨁⨁◯◯ Low |
| **Quality of life (follow-up: 1 months)** | | | | | | | | | |
| 1 | randomised trials | not serious | not serious | not serious | very serious | none | 30 | 30 | ⨁⨁◯◯ Low |
| **Duration of levelling & alignment stage (follow-up: range 4 months to 29.3 months)** | | | | | | | | | |
| 2 | randomised trials | not serious | not serious | not serious | very serious | none | 84 | 83 | ⨁⨁◯◯ Low |
| **Levelling & alignment (follow-up: 4 months)** | | | | | | | | | |
| 1 | randomised trials | not serious | not serious | not serious | very serious | none | 7 | 7 | ⨁⨁◯◯ Low |
| **Duration of working & finishing stage (follow-up: 29.3 months)** | | | | | | | | | |
| 1 | randomised trials | not serious | not serious | not serious | very serious | none | 77 | 76 | ⨁⨁◯◯ Low |
| **Duration of overall treatment (follow-up: range 1.7 years to 29.3 months)** | | | | | | | | | |
| 2 | randomised trials | serious | not serious | not serious | very serious | none | 106 | 108 | ⨁◯◯◯ Very low |
| **ABO CR-Eval** | | | | | | | | | |
| 1 | randomised trials | not serious | not serious | not serious | very serious | none | 77 | 76 | ⨁⨁◯◯ Low |
| **PAR** | | | | | | | | | |
| 1 | randomised trials | not serious | not serious | not serious | very serious | none | 77 | 76 | ⨁⨁◯◯ Low |
| **U1-PP** | | | | | | | | | |
| 1 | randomised trials | not serious | not serious | not serious | very serious | none | 77 | 76 | ⨁⨁◯◯ Low |
| **L1-MP** | | | | | | | | | |
| 1 | randomised trials | not serious | not serious | not serious | very serious | none | 77 | 76 | ⨁⨁◯◯ Low |
| **OIIRR (follow-up: 9 months)** | | | | | | | | | |
| 1 | randomised trials | not serious | not serious | not serious | very serious | none | 77 | 76 | ⨁⨁◯◯ Low |
| **ALR** | | | | | | | | | |
| 1 | randomised trials | not serious | not serious | not serious | very serious | none | 41 | 33 | ⨁⨁◯◯ Low |
| **ALL** | | | | | | | | | |
| 1 | randomised trials | not serious | not serious | not serious | very serious | none | 41 | 33 | ⨁⨁◯◯ Low |
| **Shape Change Maxilla (follow-up: 9 months)** | | | | | | | | | |
| 1 | randomised trials | very serious | not serious | not serious | very serious | none | 20 | 20 | ⨁◯◯◯ Very low |
| **Shape Change Mandible (follow-up: 9 months)** | | | | | | | | | |
| 1 | randomised trials | very serious | not serious | not serious | very serious | none | 20 | 20 | ⨁◯◯◯ Very low |
| **Loss of tooth length (follow-up: 1.7 years)** | | | | | | | | | |
| 1 | randomised trials | very serious | not serious | not serious | very serious | none | 29 | 32 | ⨁◯◯◯ Very low |
| **Prevalence of resorption (follow-up: 1.7 years)** | | | | | | | | | |
| 1 | randomised trials | very serious | not serious | not serious | very serious | none | 29 | 32 | ⨁◯◯◯ Very low |
